# New York State

**Published:** 1901-07

**Authors:** 


### NEW YORK STATE. ATTEMPTED REOPENING REGISTRATION.

The New York Assembly bill to again reopen registrations for those who had failed to comply with the laws of 1895 of that State, intended to give a period of two months for registration of those who practiced prior to 1896, and those matriculating in a reputable veterinary medical school prior to January 1, 1895, was defeated in that body.

